# Endoscopic management to different isolated sphenoid fungal pathologies: case series of a tertiary hospital

**DOI:** 10.1093/jscr/rjae844

**Published:** 2025-01-09

**Authors:** Maria Alabdulaal, Zahra AlMuhanna, Shaykhah AlNaimi, Abdulrhaman Alkhatib, Ali Almomen

**Affiliations:** Department of Otolaryngology-Head and Neck Surgery, King Fahad Specialist Hospital, Dammam 32253, Saudi Arabia; Department of Otolaryngology-Head and Neck Surgery, College of Medicine, King Fahd Hospital of the University, Al-Khobar 34445, Saudi Arabia; Department of Otolaryngology-Head and Neck Surgery, College of Medicine, King Fahd Hospital of the University, Al-Khobar 34445, Saudi Arabia; Department of Otolaryngology-Head and Neck Surgery, King Fahad Specialist Hospital, Dammam 32253, Saudi Arabia; Department of Otolaryngology-Head and Neck Surgery, King Fahad Specialist Hospital, Dammam 32253, Saudi Arabia

**Keywords:** isolated sphenoid sinus, headache, visual disturbance, nasal endoscopy, sphenoidotomy, case report

## Abstract

Isolated sphenoid sinus disease (ISSD) is a rare condition that accounts for roughly 3% of all sinusitis cases. ISSD is predominantly caused by infectious and inflammation processes, with underlying fungal pathologies. This case series aims to illustrate the endonasal endoscopic management of different isolated sphenoid fungal pathologies. We described four distinct case presentations of ISSD at a tertiary hospital in Dammam, Saudi Arabia. These cases included isolated sphenoid fungal ball, allergic fungal sinusitis, and acute invasive fungal sinusitis. The management of isolated sphenoid fungal pathologies differs depending on the type of the disease necessitating early identification and adequate treatment. The endonasal endoscopic approach is successful with minimal complications and favorable outcomes.

## Introduction

Isolated sphenoid sinus disease (ISSD) is an uncommon disorder accounting for around 3% of all sinusitis cases [1]. The etiology of ISSD varied, although it is primarily infectious and inflammatory in character [2, 3]. ISSD symptoms are nonspecific, which may cause a delay in diagnosis [4, 5]. Headache is a common presenting symptom, affecting 65.8%–69% of patients; other symptoms include facial pain, rhinorrhea, nasal congestion, post-nasal drip, vision abnormalities, and cranial nerve palsies [4, 6, 7]. Histopathological classification includes invasive and non-invasive pathologies, such as allergic fungal sinusitis (AFS), fungal ball, and acute fulminant invasive fungal sinusitis.

Furthermore, due to the anatomical location of sphenoid and its close proximity to vital structures such as the internal carotid arteries, cavernous sinus, cranial nerves II, III, IV, V1, V2, VI, pituitary gland, and dura mater, sphenoid disease can lead to serious complications like blindness, diplopia, or potentially fatal infection if left untreated [2].

Clinical suspicion, nasal endoscopy, and imaging studies using advanced imaging modalities like computed tomography (CT) and magnetic resonance imaging (MRI) are required for determining the diagnosis [5, 8].

Medical management and surgical intervention are among the treatment options that should be considered. Endoscopic surgery is the major therapeutic option for ISSD, with different techniques available based on the disease and anatomical factors [9, 10]. The surgical method chosen is determined by criteria such as illness location, scope of surgery, and anatomical configuration of the sphenoid sinus [8].

Endoscopic transnasal sphenoethmoidectomy is effective in treating various sphenoid sinus diseases [10, 11]. Providing benefits such as minor blood loss, reduced surgical time, and shorter hospital stays [12]. According to Kim *et al*. 92.2% of patients reported reduced symptoms after using this approach, which has demonstrated great outcomes [13]. Therefore, this report aims to illustrate the endonasal endoscopic management of different isolated sphenoid fungal pathologies.

## Case series

### First case: isolated sphenoid fungal ball

**Figure 1:**
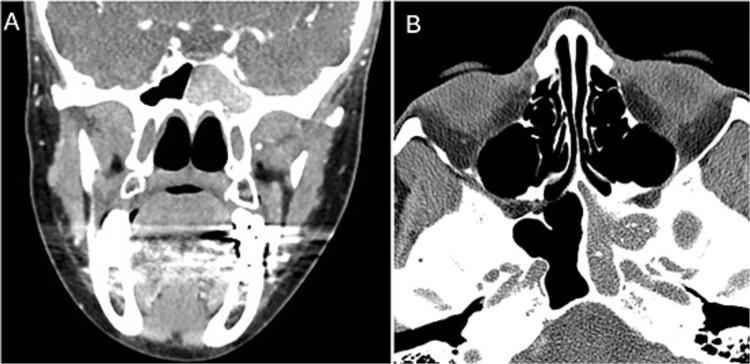
Coronal (A) and axial (B) images of a nonenhanced CT scan of the paranasal sinuses showing left sphenoid sinus complete homogenous opacification.

**Figure 2:**
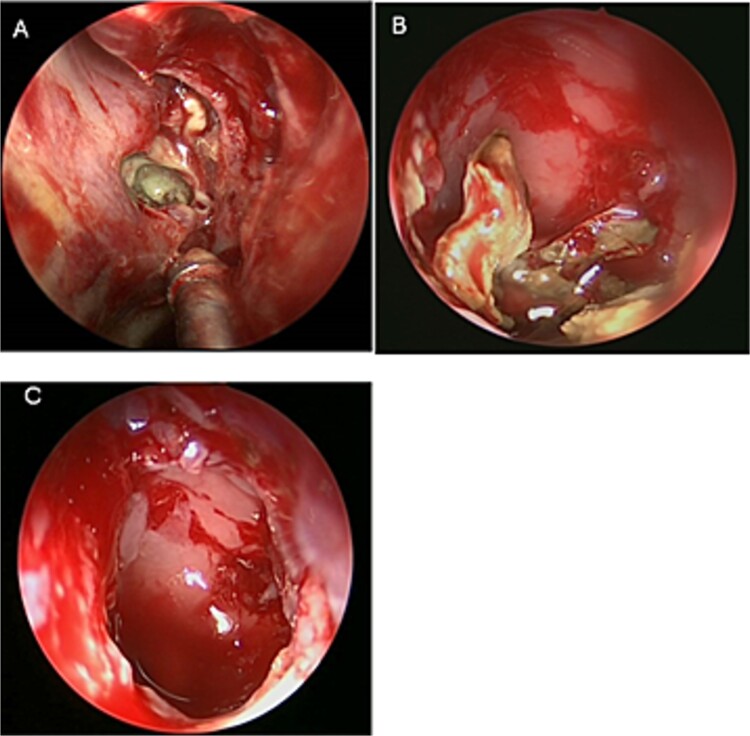
Intra-operative (A and B) endoscopic sphenoidotomy showing fungal debris, with post-operative examination (C) showing clean wide sphenoid sinus free from fungal debris.

A 36 years-old man with no previous history of medical disease presented to the otolaryngology clinic with a chief complaint of headache that was associated with pressure feeling mainly in the occipital area. He also reported a post-nasal drip. The patient underwent a non-contrast paranasal sinus CT scan (Fig. 1) that demonstrated an isolated sphenoid sinus homogenous opacification, most likely representing sphenoid fungal ball. A decision was made to proceed with endoscopic sphenoidotomy (Fig. 2) to clean and remove the debris, which confirmed the diagnosis of sphenoid sinus fungal ball.

### Second case: isolated sphenoid non-invasive allergic fungal sinusitis

**Figure 3:**
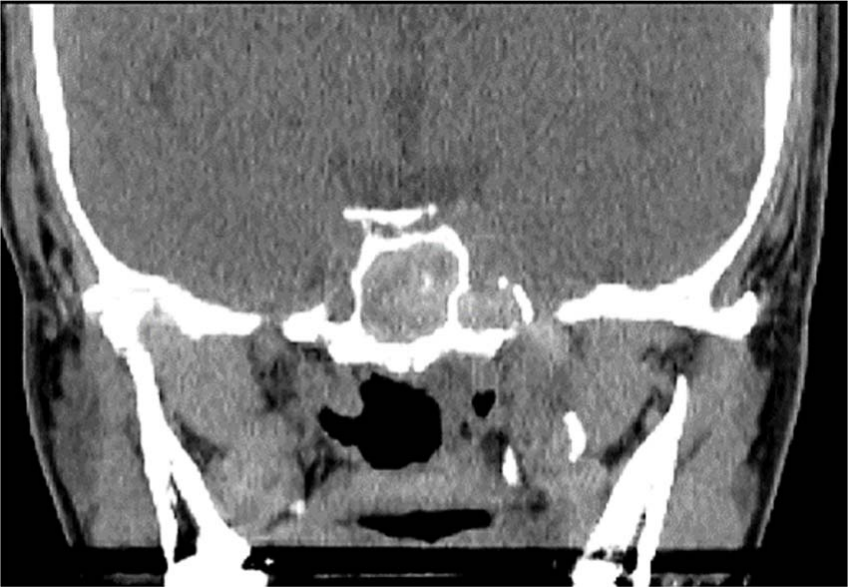
Coronal CT scan image showing complete sphenoid sinus heterogenous opacification.

**Figure 4:**
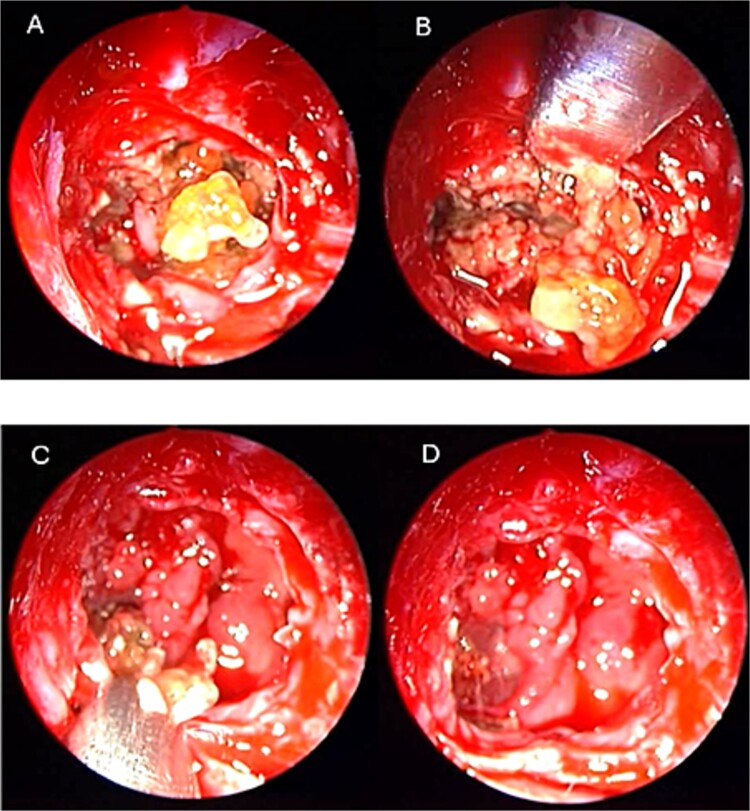
Intra-operative (a, b, and c) endoscopic sphenoidotomy showing mud and mucin, with post-operative examination (d) of right optico-carotid recess showing wide and clean sphenoid sinus.

An asthmatic 42-years-old patient who was previously medically treated for allergic rhinitis with no significant improvement. A subsequent CT scan revealed isolated sphenoid sinus disease (Fig. 3). Intra-operative endoscopic findings revealed fungal mud and mucin (Fig. 4A and B) with post-operative (Fig. 4C) endoscopic finding of widely open, clean sphenoid sinus. Further histological examination of the specimen proved the diagnosis of eosinophilic AFS.

### Third case: isolated sphenoid invasive acute fungal sinusitis

**Figure 5:**
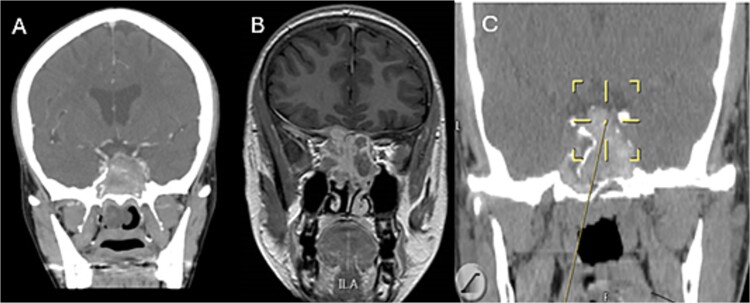
Coronal CT (A) and MRI (B) and CT image guided intra-operative (C) images of the paranasal sinuses showing aggressive sphenoid sinus disease extending to the skull base and cavernous sinus.

**Figure 6:**
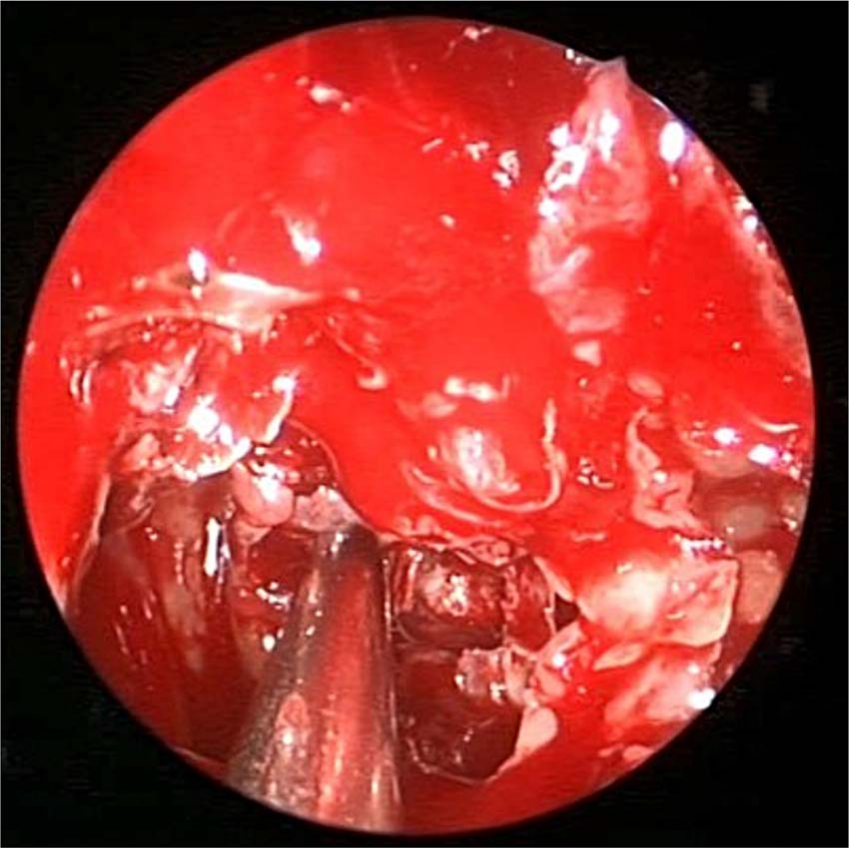
Intra-operative endoscopic view of shenoid sinus debridement of invasive fungal disease.

A 69-years-old diabetic female was referred from neurology as she complained of an acute onset severe headache, diplopia, and retro-orbital pressure. An initial CT scan with a subsequent MRI scan (Fig. 5) showed infiltrative sphenoid pathology invading both skull base and cavernous sinus given a provisional diagnosis of acute invasive fungal sinusitis. The patient was urgently taken to the operative theater to endoscopically debride sphenoid sinuses (Fig. 6). Histopathological examination confirmed the diagnosis of invasive sphenoid sinus mucormycosis.

## Discussion

The isolated sphenoid sinus can host a variety of fungal infections, both invasive and non-invasive. Understanding the variations between these conditions is essential for accurate diagnosis and treatment.

### Non-invasive sphenoid sinus fungal ball (Mycetoma)

Sphenoid sinus fungal ball (SSFB) is relatively common, contributing to 11%–13% of all paranasal sinus fungal ball cases [14]. It primarily affects elderly individuals, with a significant preference for women [15]. The clinical manifestations of SSFB can be difficult to diagnose duo to its unspecific symptoms, which frequently include nasal problems and headaches [16]. Also, visual complications tend to increase in older age, diabetes mellitus, and the coexistence of sphenoid sinus wall anomalies [17]. Usually, the diagnosis is made by CT, which shows distinctive characteristics such opacity, central microcalcification, along with potential alteration to the bone wall. This finding is subsequently verified by histological analysis, which reveals fungal elements without tissue invasion [3]. The standard surgical approach for SSFB is endoscopic sphenoidotomy, which involves removing any fungal debris completely and this has been shown to be curative [18]. In addition, antifungal drugs are usually not needed after surgery if there is no bone or mucosal invasion. After surgical intervention, there is a favorable overall prognosis and a low risk of recurrence [4].

### Isolated sphenoid non-invasive allergic fungal sinusitis

AFS is a distinct form of chronic rhinosinusitis characterized In contrast to other forms of chronic rhinosinusitis, AFS is typified by an immunoglobulin E (IgE)-mediated reaction to fungal pathogens in the paranasal sinuses [19]. Specific criteria, such as the lack of tissue invasion, positive fungal staining or culture, and the presence of distinctive allergic mucin with fungal hyphae, are necessary for the diagnosis of AFS.

When it comes to diagnosis, imaging is essential. CT scans reveal abnormalities and areas of hyperattenuation within affected sinuses. AFS pathogenesis involves a complicated interaction of type I hypersensitivity, increased IgE levels, and perhaps *Staphylococcus aureus* superantigens [20]. Frequently, post-operative oral corticosteroids are used after the surgical removal of diseased tissue and allergic mucin. Although systemic antifungals are not advised in AFS, allergen immunotherapy may help reduce relapses [21].

### Isolated sphenoid invasive acute fulminant fungal sinusitis

Invasive acute fulminant fungal sinusitis (IAFFS) is a rare but life-threatening illness that predominantly affects immunocompromised people, especially individuals with diabetes or going through chemotherapy. This highlights the necessity for a high index of suspicion in all patient categories [22]. It is distinguished by fast fungal invasion of the mucosa, submucosa, and ultimately the bone, resulting in necrosis. If left untreated, this aggressive invasion may result in serious problems such as cavernous sinus thrombosis, loss of vision, and potentially death. IAFFS patients frequently experience vague symptoms including headaches and ocular abnormalities, and the disease’s clinical manifestations can be deceptively modest [23]. Predicting early changes and assessing the severity of disease depend primarily on diagnostic imaging, particularly by MRI [24]. However, the absence of adequate sensitivity and specificity in present diagnostic techniques emphasizes the necessity for further developed diagnostic tools. Usually, rigorous surgical debridement and antifungal medication are used in combination for treatment. Despite these measures, the prognosis for sphenoid sinus IAFFS is still poor, particularly when there is intracranial extension [25].

## Conclusion

Non-invasive forms of isolated sphenoid sinus fungal diseases such as fungal ball and AFS benefit mainly from surgical procedure, nevertheless the latter necessitate continuing medical treatment given its chronic nature. On the other hand, invasive forms, like acute fulminant fungal sinusitis, require surgical and medical management in a timely manner. Further studies must focus on creating more reliable diagnostic indicators and novel strategies for treatment. Recognizing the differences in pathology is crucial for prompt and proper treatment to reduce complications and enhance the outcome of patients.

## References

[1] Perkasa MF . Isolated fungal sinusitis of the sphenoid sinus - a case report. Gac Med Caracas 2023;131:654–8. 10.47307/GMC.2023.131.s4.21.

[2] Chao CC , Lin YT, Lin CF, et al. The clinical features of endoscopic treated isolated sphenoid sinus diseases. J Formos Med Assoc 2021;120:1554–62. 10.1016/j.jfma.2020.11.005. 33246742

[3] Marcolini TR , Safraider MC, Socher JA, et al. Differential diagnosis and treatment of isolated pathologies of the sphenoid sinus: retrospective study of 46 cases. Int Arch Otorhinolaryngol 2015;19:124–9. 10.1055/s-0034-1397337. 25992167 PMC4399171

[4] Friedman A , Batra PS, Fakhri S, et al. Isolated sphenoid sinus disease: Etiology and management. Otolaryngol-Head Neck Surg 2005;133:544–50. 10.1016/j.otohns.2005.04.023. 16213927

[5] Sethi DS . Isolated sphenoid lesions: diagnosis and management. Otolaryngol - Head Neck Surg 1999;120:730–6. 10.1053/hn.1999.v120.a89436. 10229601

[6] Kanoh N , Xu R, Wang ZM, et al. Isolated sphenoid sinus disease: an analysis of 122 cases. Ann Otol Rhinol Laryngol 2002;111:323–7. 10.1177/000348940211100407. 11991583

[7] Martin TJ , Smith TL, Smith MM, et al. Evaluation and surgical management of isolated sphenoid sinus disease. Arch Otolaryngol - Head Neck Surg 2002;128:1413–9. 10.1001/archotol.128.12.1413. 12479731

[8] Tang IP , Brand Y, Prepageran N. Evaluation and treatment of isolated sphenoid sinus diseases. Curr Opin Otolaryngol Head Neck Surg 2016;24:43–9. 10.1097/MOO.0000000000000218. 26575516

[9] Nour YA , Al-Madani A, El-Daly A, et al. Isolated sphenoid sinus pathology: Spectrum of diagnostic and treatment modalities. Auris Nasus Larynx 2008;35:500–8. 10.1016/j.anl.2007.10.011. 18242904

[10] Socher JA , Cassano M, Filheiro CA, et al. Diagnosis and treatment of isolated sphenoid sinus disease: a review of 109 cases. Acta Otolaryngol 2008;128:1004–10. 10.1080/00016480701793735. 19086308

[11] Strek P , Zagólski O, Składzień J, et al. Endoscopic surgical treatment of patients with isolated sphenoid sinus disease. Otolaryngol Pol 2007;61:254–9. 17847777 10.1016/S0030-6657(07)70422-5

[12] Hadar T , Yaniv E, Shvero J. Isolated sphenoid sinus changes - history, CT and endoscopic finding. J Laryngol Otol 1996;110:850–3. 10.1017/S0022215100135145. 8949295

[13] Kim SW , Kim DW, Kong IG, et al. Isolated sphenoid sinus diseases: report of 76 cases. Acta Otolaryngol 2008;128:455–9. 10.1080/00016480701762466. 18368582

[14] Kim JS , Kim BK, Hong SD, et al. Clinical characteristics of sphenoid sinus fungal ball patients with visual disturbance. Clin Exp Otorhinolaryngol 2016;9:326–31. 10.21053/ceo.2015.01571. 27136367 PMC5115146

[15] Eloy P , Grenier J, Pirlet A, et al. Sphenoid sinus fungall ball: a retrospective study over a 10-year period. Rhinology 2013;51:181–8. 10.4193/Rhino12.114. 23671900

[16] Leroux E , Valade D, Guichard JP, et al. Sphenoid fungus balls: clinical presentation and long-term follow-up in 24 patients. Cephalalgia 2009;29:1218–23. 10.1111/j.1468-2982.2009.01850.x. 19811505

[17] Chen F , Shao Y, Huang Q, et al. Visual function loss in fungal sphenoid sinusitis: clinical characteristics and outcomes. Sci Rep 2024;14:8649–9. 10.1038/s41598-024-59107-2. 38622183 PMC11018747

[18] Almomen A , Albaharna H, Al Shakhs A, et al. Sphenoid sinus fungal ball, a tertiary hospital experience. Int J Otorhinolaryngol 2020;6:16. 10.11648/j.ijo.20200601.14.

[19] Walter E , McKean EL, Camelo-Piragua SI, et al. Catastrophic allergic fungal sinusitis: a report of two cases. J Neuro-Ophthalmology 2020;40:507–13. 10.1097/WNO.0000000000000833. 31609841

[20] Doellman MS , Dion GR, Weitzel EK, et al. Immunotherapy in allergic fungal sinusitis: the controversy continues. A recent review of literature. Allergy Rhinol 2013;4:e32–5. 10.2500/ar.2013.4.0045. PMC3679565 23772324

[21] Hall AG , DeShazo RD. Immunotherapy for allergic fungal sinusitis. Curr Opin Allergy Clin Immunol 2012;12:629–34. 10.1097/ACI.0b013e328357a233. 23095911

[22] Chopra H , Dua K, Malhotra V, et al. Invasive fungal sinusitis of isolated sphenoid sinus in immunocompetent subjects. Mycoses 2006;49:30–6. 10.1111/j.1439-0507.2005.01170.x. 16367816

[23] Epstein VA , Kern RC. Invasive fungal sinusitis and complications of rhinosinusitis. Otolaryngol Clin North Am 2008;41:497–524. 10.1016/j.otc.2008.01.001. 18435995

[24] Howells RC , Ramadan HH. Usefulness of computed tomography and magnetic resonance in fulminant invasive fungal rhinosinusitis. Am J Rhinol 2001;15:255–61. 10.1177/194589240101500407. 11554658

[25] Lee DH , Yoon TM, Lee JK, et al. Invasive fungal sinusitis of the sphenoid sinus. Clin Exp Otorhinolaryngol 2014;7:181–7. 10.3342/ceo.2014.7.3.181. 25177433 PMC4135153

